# Assaying kinase activity of the TPL-2/NF-κB1 p105/ABIN-2 complex using an optimal peptide substrate

**DOI:** 10.1042/BCJ20170579

**Published:** 2018-01-11

**Authors:** Sandra Kümper, Thorsten Gantke, Chao-Sheng Chen, Yasmina Soneji, Michael J. Pattison, Probir Chakravarty, Svend Kjær, Daniel Thomas, Carl Haslam, Bill J. Leavens, David House, David J. Powell, Steven C. Ley

**Affiliations:** 1 Crick-GSK Biomedical LinkLabs, GlaxoSmithKline, Stevenage SG1 2NY, U.K.; 2 RD Platform Technology & Science, GlaxoSmithKline, Stevenage SG1 2NY, U.K; 3 Immune Cell Signalling Laboratory, The Francis Crick Institute, London NW1 1AT, U.K.; 4 Bioinformatics and Biostatistics, The Francis Crick Institute, London NW1 1AT, U.K.; 5 Structural Biology, Science Technology Platforms, The Francis Crick Institute, London NW1 1AT, U.K.

**Keywords:** high-throughput assay, inflammation, kinase, TPL-2

## Abstract

The MKK1/2 kinase tumour progression locus 2 (TPL-2) is critical for the production of tumour necrosis factor alpha (TNFα) in innate immune responses and a potential anti-inflammatory drug target. Several earlier pharmaceutical company screens with the isolated TPL-2 kinase domain have identified small-molecule inhibitors that specifically block TPL-2 signalling in cells, but none of these have progressed to clinical development. We have previously shown that TPL-2 catalytic activity regulates TNF production by macrophages while associated with NF-κB1 p105 and ABIN-2, independently of MKK1/2 phosphorylation via an unknown downstream substrate. In the present study, we used a positional scanning peptide library to determine the optimal substrate specificity of a complex of TPL-2, NF-κB1 p105 and ABIN-2. Using an optimal peptide substrate based on this screen and a high-throughput mass spectrometry assay to monitor kinase activity, we found that the TPL-2 complex has significantly altered sensitivities versus existing ATP-competitive TPL-2 inhibitors than the isolated TPL-2 kinase domain. These results imply that screens with the more physiologically relevant TPL-2/NF-κB1 p105/ABIN-2 complex have the potential to deliver novel TPL-2 chemical series; both ATP-competitive and allosteric inhibitors could emerge with significantly improved prospects for development as anti-inflammatory drugs.

## Introduction

The pro-inflammatory cytokine tumour necrosis factor alpha (TNFα) plays an important role in the pathogenesis of multiple inflammatory diseases and biological agents that block TNF activity have been successfully used to treat rheumatoid arthritis, Crohn's disease, psoriatic arthritis and ankylosing spondylitis [1]. However, such antibody drugs are expensive, require repeated injection and are only effective in a fraction of patients. There is, therefore, a need for more effective, less expensive and, ideally, orally active drugs to suppress the production of TNF by the innate immune system [2].

In inflammatory responses to pathogen infection, macrophages are a major source of TNF, triggered by stimulation of pattern recognition receptors. These include Toll-like receptors (TLRs) such as TLR4, which respond to lipopolysaccharide (LPS), a surface component of Gram-negative bacteria [3]. Tumour progression locus 2 (TPL-2) kinase is a critical regulator of TNF production by TLR4-stimulated macrophages. Production of TNF after LPS injection is substantially reduced in TPL-2-deficient mice, which are resistant to septic shock induction [4]. Analyses of *Tpl2*^−/−^ mice have also indicated key pro-inflammatory roles for TPL-2 in many other disease models, including inflammatory bowel disease, pancreatitis, liver fibrosis and experimental autoimmune encephalomyelitis [5–7]. The importance of TPL-2 in inflammation and its critical role in regulating production of the key pro-inflammatory cytokine TNF have made TPL-2 an attractive target for the development of novel anti-inflammatory drugs [8].

TPL-2 is a MKK 1/2 kinase, which mediates activation of ERK1/2 MAP kinases by TLR4 (and all other TLRs), as well as the receptors for TNF and IL-1β, in macrophages [5]. Several pharmaceutical companies have carried out screens using the isolated TPL-2 kinase domain and MKK1 substrate to identify inhibitors that block TPL-2 activation of ERK1/2 in cultured macrophages [8,9]. However, none of these have progressed to clinical trial, likely reflecting the challenge of producing ATP-competitive inhibitors with the appropriate physicochemical characteristics required for drug development. An alternative screening paradigm utilizing TPL-2 in its native context could provide a tractable route to clinical development.

In unstimulated cells, TPL-2 forms a stoichiometric complex with NF-κB1 p105 and the ubiquitin-binding protein ABIN-2, which are both required for the maintenance of TPL-2 steady-state levels. Binding to p105 also prevents TPL-2 phosphorylation of MKK1/2 [10]. Consequently, TPL-2 activation of the ERK1/2 MAP kinase pathway requires TPL-2 release from p105, which is triggered by IκB kinase (IKK)-induced proteolysis of p105 by the proteasome [11–13]. Surprisingly, we have recently found that TPL-2 catalytic activity regulates TNF production by macrophages independently of IKK-induced p105 proteolysis and ERK1/2 activation, while still associated with p105 and ABIN-2 [13]. While the target substrate that controls TNF production has not been identified, these results indicate that the TPL-2/NF-κB1 p105/ABIN-2 complex actively signals and that small-molecule inhibitors targeting this complex should block TNF production and possibly other pro-inflammatory functions of TPL-2.

In the present study, we used a positional scanning peptide library to identify the optimal peptide phosphorylation motif of recombinant TPL-2/NF-κB1 p105/ABIN-2 complex [14]. Using a synthetic peptide based on this optimal motif, we developed a high-throughput screening assay and used this to demonstrate that the TPL-2 complex has a substantially altered sensitivity to existing TPL-2 inhibitors compared with the isolated TPL-2 kinase domain. These results suggest that inhibitor screens using the TPL-2/NF-κB1 p105/ABIN-2 complex instead of the isolated kinase domain may yield novel series of TPL-2 inhibitors.

## Experimental

### Plasmids, peptides and inhibitors

Expression plasmids (pcDNA3 vector; Life Technologies, Inc.) encoding hexahistidine-tagged (His_6_) TPL-2 and TPL-2^D270A^ (NCBI RefSeq: NP_001231063.1), haemagglutinin (HA)-tagged NF-κB1 p105 and C-terminally StrepII-tagged ABIN-2 (NCBI RefSeq NP_077285.3) have been described previously [14,15]. His_6_–TPL-2^30–404^ was produced by PCR amplification and subcloned into the pFastBac vector (Invitrogen). The pcDNA3 plasmid encoding HA-VP35 of the Ebola virus species *Zaire ebolavirus* (Mayinga isolate) has been described [16].

MKK1 and TPL-2tide peptides (biotinylated at their C-terminus) were synthesized and HPLC-purified (95% purity) by GL Biochem (Shanghai, China). The MKK1 peptide, which corresponded to the activation loop of MKK1 (YAGQLIDSMANSFVGTAGKK), has been previously described [17]. TPL-2tide and pTPL2tide used in the mass spectrometry assay were synthesized by Cambridge Research Biochemicals. TPL-2tide (YADDDDDSFLWNAGKK) was an optimized TPL-2 peptide substrate predicted by the optimal sequence motif. The S5 peptide (GAFRSAIRRLAARRR-acid) was optimized from an IMAP peptide library screen (Molecular Devices), which identified the parent peptide, FAM-GTFRSSIRRLSTRRR-acid, as the most efficient substrate for the isolated TPL-2 kinase domain. The sequence was mutated at four of the five Ser/Thr residue positions to generate daughter peptides with only a single phosphorylation site. Subsequent testing identified the S5 version to be the most efficient TPL-2 kinase domain substrate.

Abbott C41, Wyeth C1, Wyeth C34 and Wyeth C2p TPL-2 inhibitors, as described [18–21], were synthesized according to published procedures. Proton NMR and LC–MS spectra were in accordance with published data.

### Expression of recombinant TPL-2

HEK293 cells (QBI293A cells, Quantum Biotechnologies) were grown in suspension cultures as described previously [14]. For expression of recombinant TPL-2/NF-κB1 p105/ABIN-2 complex, cells were pelleted by centrifugation and resuspended at a density of 4.0 × 10^6^ cells/ml in standard culture medium [Pro293s-CDM medium (Lonza), supplemented with 1.5% foetal bovine serum, 2 mM l-glutamine, 50 U/ml penicillin and 50 U/ml streptomycin]. DNA complexed with linear polyethyleneimine (25 kDa) at a ratio of 3 : 1 (w/w) was then added to a final concentration of 2 μg DNA/ml. After 5 h, cell density was adjusted to 2.0 × 10^6^ cells/ml and cells were cultured for a total of 72 h. Cells were lysed in buffer A [50 mM Tris–HCl (pH7.5), 0.5% IGEPAL CA-630, 150 mM NaCl, 10 mM imidazole, 10 mM Na-fluoride, 1 mM Na-pyrophosphate, 10 mM β-glycerophosphate, 0.5 mM tris(2-carboxyethyl)phosphine (TCEP) and 10% glycerol plus Complete™ Protease Inhibitor Cocktail (Roche)].

TPL-2^30–404^ protein was produced following a published methodology [9]. Sf9 cells were co-transfected with pFastbac virus DNA for baculovirus generation. Protein expression was carried out in 5-l cell cultures of Sf9 cells with plaque-purified viruses. Compound 1 (10 µM; [9]) was added to the cell cultures at 24 h to improve recombinant TPL-2^30–404^ protein yields. Cells were harvested 72 h after infection, pellets were snap-frozen and stored at −80°C.

### Protein purification

For peptide library screening, recombinant His_6_–TPL-2/ABIN-2–StrepII/HA–p105 complex was purified by sequential affinity purification. Centrifuged lysates were incubated with Ni-NTA (Ni^2+^-nitrilotriacetate)-agarose (Qiagen) for 60 min, washed in DM buffer [50 mM Tris–HCl (pH 7.5), 1.8 mM decyl β-d-maltopyranoside (DM), 150 mM NaCl, 10 mM imidazole, 10 mM Na-fluoride, 1 mM Na-pyrophosphate, 10 mM β-glycerophosphate, 0.5 mM TCEP and 10% glycerol supplemented with protease inhibitors]. Bound protein was eluted with DM buffer supplemented with 200 mM imidazole. After adding EDTA to a final concentration of 1 mM, eluates were subsequently incubated with StrepTactin Sepharose (GE Healthcare) to specifically purify StrepII–ABIN-2-containing TPL-2 complexes, washed extensively in DM buffer and bound protein eluted with 2.5 mM desthiobiotin. To remove desthiobiotin, eluted His_6_–TPL-2 was then loaded on to an Ni^2+^-charged HisTrap HP column (GE Healthcare), followed by extensive washing and elution with 200 mM imidazole. Sample purity of the isolated His_6_–TPL-2/ABIN-2–StrepII/HA–p105 complex was quantified by measuring the infrared fluorescence of Coomassie-stained protein SDS–PAGE gels (Odyssey Infrared Imaging System, LI-COR Biosciences) and shown to be >90%.

A modified three-step purification method for the recombinant His_6_–TPL-2/HA–p105/ABIN–2-StrepII complex was used for mass spectrometric assays of TPL-2tide phosphorylation. Centrifuged lysates were applied to a StrepTRAP HP column (GE Healthcare) and pre-equilibrated with Buffer A, at 1 ml/min. Bound protein was eluted with DM buffer containing 2.5 mM desthiobiotin and loaded on to a Superdex 200 size-exclusion column (GE Healthcare) and then pre-equilibrated with Buffer B [50 mM Tris–HCl (pH 7.5), 1.8 mM DM, 150 mM NaCl, 0.5 mM TCEP and 10% glycerol]. High molecular mass fractions containing TPL-2 complex were pooled and applied to a HiTrap Q ion-exchange column (GE Healthcare). The sample was eluted with a 1 M NaCl 10 column volume gradient in Buffer B. Sample purity (>90%) was assessed by Coomassie Brilliant Blue staining of SDS–PAGE gels.

For purification of His_6_–TPL-2^30–404^, Sf9 cell pellets were resuspendend in lysis buffer [50 mM Tris–HCl (pH 8), 3 mM TCEP, 400 mM NaCl, 10% glycerol, 20 mM imidazole and 0.05% Triton X-100, supplemented with protease inhibitors]. Compound 1 [9] (dissolved in DMSO) was added to give a final concentration of 20 µM, followed by homogenization of the cell lysate. Cleared lysates were loaded on to a HisTrap HP column (GE Healthcare), pre-equilibrated with lysis buffer. After extensive washing with lysis buffer supplemented with 200 mM NaCl, His_6_–TPL-2^30–404^ protein was eluted with a 20–300 mM imidazole gradient. Fractions containing His_6_–TPL-2^30–404^ were pooled and loaded onto a Superdex 75 size-exclusion column (GE Healthcare), pre-equilibrated with a buffer containing 50 mM Tris–HCl (pH 8), 150 mM NaCl, 0.5 mM TCEP and 10% glycerol. Fractions containing His_6_–TPL-2^30–404^ were pooled and concentrated by ultrafiltration to 5 mg/ml. Sample purity, assessed by Coomassie Brilliant Blue staining of SDS–PAGE gels, was ∼90%.

### Peptide library screening

The positional scanning synthetic peptide library described by Hutti et al. [22] was purchased from AnaSpec, U.S.A. (ANA62017-1 and ANA62335). Peptides were dissolved in de-gassed DMSO and the concentration of each peptide mix was adjusted to 0.6 mM by the addition of Tris–HCl (pH 7.5). Peptide library screening was performed by incubation of 30 nM recombinant TPL-2 complex with 50 µM peptide substrate in kinase buffer [50 mM Tris–HCl (pH 7.5), 150 mM NaCl, 0.01% Brij-35, 5 mM MnCl_2_, 2 mM DTT, 10 µM ATP and 3 nCi/µL [γ-^32^P]ATP] at 30°C for 1 h. Control labelling experiments were performed in the presence of 10 µM C34 TPL-2 inhibitor [21]. After incubation, reactions were transferred onto streptavidin-coated membranes (SAM2, Promega, U.S.A.) using a 384-well 2 µL slot pin replicator (V&P Scientific, U.S.A.). Membranes were washed two times 15 min in TBS/1% SDS and then overnight in the same buffer. Membranes were washed a further three times 15 min with 2 M NaCl/1% H_3_PO_4_ before washing briefly with water and 96% EtOH. Membranes were air-dried and peptide labelling was quantified by phosphorimaging (Storm PhosphorImager, GE Healthcare, U.K.) using the ImageQuant TL data analysis software.

Spot intensities from three replicate experiments were quantified using the ImageQuant TL Data Analysis software (Bio-Rad, U.S.A.) and mean values were calculated. The intensity of each spot was normalized by the mean intensity of all spots in a single position relative to the central phosphoacceptor site to calculate selectivity values (*χ_i_*) for each amino acid in every position. Selectivity values reflected positive or negative selection of an amino acid at one position. These ranged from 0 to 20, indicating either full negative or positive selection, respectively. Selectivity values of 1.0 indicated neutral selectivity. Position-specific scoring matrices (PSSMs) describing amino acid selectivity at all positions from P − 5 to P + 4 were assembled and used to analyze linear sequences with Scansite 2.0 (http://www.scansite.mit.edu) [23]. Because of possible overestimation of serine or threonine preference due to the presence of additional photoacceptor residues in peptides with serine or threonine at fixed positions, a second PSSM was assembled which disregards all positive preferences for either serine or threonine (termed ‘No Ser/Thr’ motif). Scansite analyses linear sequences by deriving bit scores (*s*) for each of seven positions (*i*) on either sides of serines and threonines present in a sequence of interest:

$$S_{i\;}=\frac{\ln(\chi_{i})}{\ln2}$$

A raw sequence score (*S*_raw_) is then given by

$$S_{\mathrm{raw}\;}=\sum \frac{s_{i}}{i}(i\;=1,\leq 15)$$

which is compared with the best possible score (*S*_opt_) to calculate a final sequence score

$$S_{f}=\frac{\left(S_{\mathrm{opt}}-S_{\mathrm{raw}}\right)}{S_{\mathrm{opt}}}$$

Final sequence scores describe how closely a linear sequence matches the optimal selectivity motif described by the PSSM. Accordingly, good sequence matches will yield scores approximating *S_f_* = 0, while neither positive nor negative selection for a given sequence (*S*_raw_ = 0) will result in *S_f_* = 1.0.

### Kinase assays

Phosphorylation of specific peptides by the TPL-2/p105/ABIN-2 complex was measured using either a radioactive filter binding assay or by mass spectrometry.

#### Filter binding

Biotinylated peptides (50 µM) were phosphorylated with 30 nM recombinant TPL-2 complex in kinase buffer [50 mM Tris–HCl (pH7.0), 0.03% Brij-35, 2 mM DTT, 5 mM β-glycerophosphate, 5 mM MnCl_2_ and 5% DMSO], supplemented with 0.1 mM ATP and 0.02 µCi/µl [γ-^32^P]ATP at 30°C for the indicated times. Reactions were stopped by the addition of EDTA. Reaction mixes were subjected to ultrafiltration (10 kDa cutoff; AcroPrep 384 Filter Plate, PALL Lifer Sciences, U.S.A.) to separate substrates and autophosphorylated kinase, before spotting onto streptavidin-coated membranes (SAM2, Promega). Membranes were extensively washed in TBS/1% SDS, 2 M NaCl and 2 M NaCl/1% H_3_PO_4_, and peptide phosphorylation was quantified by phosphorimaging (Storm PhosphorImager, GE Healthcare, U.K.) using the ImageQuant TL data analysis software.

#### RapidFire**™** mass spectrometry

10 mM stock solutions of TPL-2tide peptide (30% (v/v) DMSO in H_2_O) and S5 peptide (H_2_O) were prepared and used as substrates. Assays were performed in clear 384-well V-bottom polypropylene plates (Greiner #781280). The final assay volume was 10 µL in assay buffer [10 mM Tris–HCl (pH 7.2), 10 mM MgCl_2_, 0.05% NaN_3_, 0.01% Tween-20 and 1 mM DTT; Molecular Devices #R8155). Enzyme and peptide concentrations are given in the figure legends. Enzymes were serially diluted, and a substrate solution containing final concentrations of 100 µM peptide and 400 µM ATP (TPL-2tide) and 10 µM peptide and 300 µM ATP (S5 peptide) were added.

To compare compound potencies towards TPL-2^30–404^ and TPL-2/p105/ABIN-2 using TPL-2tide, enzyme solutions were added to each well of a 384-well plate and compounds were dispensed using a HP digital dispenser D-300 (Software version 3.0.1). After a 10 min pre-incubation with compound, a substrate solution containing final concentrations of 100 µM peptide and 400 µM ATP were added. All assays were run for 30 min at room temperature. Reactions were quenched by the addition of 50 µL of 1% (v/v) formic acid (aqueous).

Quenched plates were sealed and centrifuged at 1550×***g*** for 10 min before analysis on a RapidFire™ high-throughput solid-phase extraction (SPE) system (Agilent Technologies, MA, U.S.A.). The sample was aspirated directly from each well of quenched assay plates for 600 ms and loaded onto the RapidFire micro-scale solid-phase C4 (Agilent type A) extraction cartridge to remove buffer salts with RapidFire buffer A [0.1% (v/v) formic acid (aqueous)] for 3 s at a flow rate of 1.5 ml/min. Analytes were then co-eluted directly into the mass spectrometer source for a further 3 s using RapidFire buffer B [0.1% (v/v) formic acid in 80% acetonitrile (aqueous)] at a flow rate of 1.25 ml/min. This was followed by a 500 ms re-equilibration step giving a total elution cycle of 7.1 s.

TPL-2tide and the phosphorylated TPL-2tide were monitored on a Sciex API 6500 triple quadrupole mass spectrometer (Applied Biosystems, Concord, Ontario, Canada) in positive electrospray ionization (ESI) mode following multiple reaction monitoring (MRM) transitions initially at (Q1 mass/Q3 mass) 913.7/91.0 Da, 953.6/904.3 Da with a scan rate of 50 ms/transition, respectively. These masses relate to the doubly charged peptide species. S5 peptide and the phosphorylated S5 peptide were monitored on a Sciex API 4000 triple quadrupole mass spectrometer (Applied Biosystems, Concord, Ontario, Canada) in positive ESI mode following MRM transitions initially at (Q1 mass/Q3 mass) 586.2/120 Da, 612.9/120 Da with a scan rate of 50 ms/transition.

### Data analysis

Peaks were integrated using the RapidFire integrator software to give peak area counts. Linear or non-linear regression fits were carried using GraFit version 7.0.3 (Erithacus Software Ltd, Surrey, U.K.), using peak area counts of phosphorylated TPL-2tide and S5 peptide for data analysis. To compare compound potencies towards different enzymes, the following formula was used:

$$\mathrm{TPL}-2\;\mathrm{activity}\text{\%}=\left(\frac{a}{d}\right)\times 100$$

In this equation, *a* is the product count at each individual inhibitor concentration and *d* is the uninhibited product count value. IC_50_ data were calculated using the following equation:

$$y=\frac{\mathrm{Range}}{1+(x/IC_{50})}+\mathrm{Background}$$

‘Range’ is the fitted uninhibited value minus the Background, and *s* is the Hill coefficient slope factor. The equation assumes that the *y* value falls with increasing *x*. For each compound, multiple replicates were run for each experiment and the experiment was repeated four to five times. The IC_50_ values and standard errors were generated by combining the data of all experiments.

## Results and discussion

### Primary phosphorylation sequence preference of the TPL-2/NF-κB p105/ABIN-2 complex

TPL-2 induces TNF production in LPS-stimulated macrophages independently of IKK-induced p105 phosphorylation [13]. Consequently, this does not involve TPL-2 phosphorylations of the MAP 2-kinases MKK1, MKK2, MKK3 or MKK6, which are blocked by *Nfkb1*[SSAA] mutation [24,25]. Furthermore, these results imply that the key substrate regulating the production of TNF is phosphorylated by TPL-2/ABIN-2/NF-κB1 p105 complex since *Nfkb1*[SSAA] mutation prevents the release of TPL-2 from p105 and ABIN-2. In an attempt to develop an assay for the catalytic activity of complexed TPL-2, we used a positional scanning synthetic peptide library to determine the primary sequence preference for phosphorylation by TPL-2 associated with p105 and ABIN-2.

The peptide library consisted of 198 distinct peptide mixtures [23] (Supplementary Figure S1). Each mixture contained an equimolar mixture of peptides with Ser or Thr in a fixed central position. In addition, one of the 20 amino acids, or phospho-Thr or phospho-Tyr, was fixed at one position relative to the central Ser/Thr, while all other positions contained an equimolar mixture of all other amino acids excluding Ser, Thr and Cys. Ser or Thr residues in the fixed positions in addition to the central phosphoacceptor site would obscure the kinase preference for these residues, as it could not be determined which residue was phosphorylated by autoradiography readout. Within the peptide library, all amino acids were tested at nine positions relative to a central phosphoacceptor. Peptide sequences were flanked by additional amino acids on either side to enhance peptide solubility and facilitate peptide handling during library preparation. A C-terminal biotin tag allowed substrate capture on streptavidin-coated membranes following the reaction.

**Figure 1.**
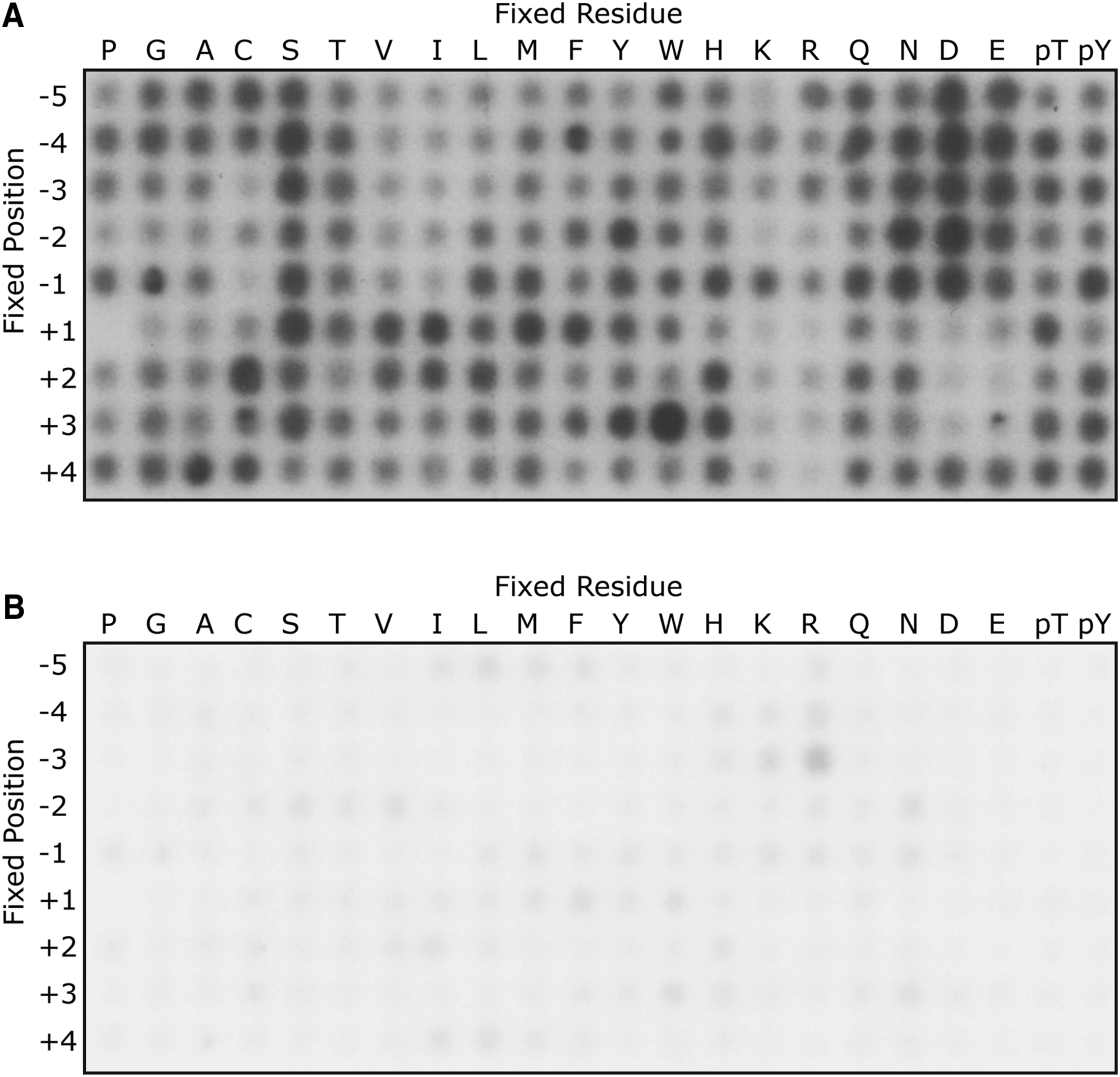
The primary amino acid sequence specificity of TPL-2. The peptide library comprised 198 individual biotinylated peptide mixtures. Each peptide contained a central phosphoracceptor Ser or Thr, flanked by degenerate positions, comprising an equimolar mixture of the 17 amino acids, excluding Cys, Ser and Thr. In each peptide, one position was fixed (fixed residue) with one of the 20 naturally occurring unmodified amino acids, phosphor-Thr (pT) or phosphor-Tyr (pY). Peptides were incubated with 30 nM recombinant TPL-2/NF-κB1 p105/ABIN-2 complex at a final substrate concentration of 50 µM at 30°C for 1 h. Assays were performed in TPL-2 kinase buffer plus 10 µM ATP and 3 nCi/µL [γ-^32^P]ATP in the absence (**A**) or presence (**B**) of C34 TPL-2 inhibitor (10 µM). Following incubation, reactions were transferred onto SAM2 membranes, which were then washed extensively. Incorporation of ^32^P into peptides was quantified by phosphorimaging.

The purified TPL-2/NF-κB1 p105/ABIN-2 complex was incubated for 1 h with the peptide mixtures of the oriented degenerate peptide library in kinase buffer containing [γ^32^P]ATP. Reaction mixtures were then transferred to streptavidin capture membranes, washed and phosphorylation visualized by autoradiography. Radioactivity of varying intensity was detected in spots of most reactions (Figure 1A). The addition of 10 µM C34 TPL-2 inhibitor [21] to the reactions significantly reduced signal intensity, confirming that the kinase assays were detecting TPL-2 activity (Figure 1B). The addition of 10 µM staurosporine, a potent protein kinase inhibitor that does not affect TPL-2 catalytic activity [26], did not affect peptide phosphorylation (data not shown).

Analysis of spot intensities revealed that TPL-2 positively selected for acidic residues in all ‘minus’ positions from P − 5 to P − 1, with a preference for Asp over Glu. TPL-2 had little activity towards peptides containing fixed hydrophobic or positively charged amino acids at these positions. Peptides containing negatively or positively charged amino acids at P + 1 to P + 3 were minimally labelled by TPL-2, and peptides with a Pro at P + 1 were not labelled. Hydrophobic amino acids at P + 1 to P + 3 were preferentially phosphorylated, with the strongest positive selection for Trp at P + 3. Strong phosphorylation was observed for peptides with fixed Ser residues at most positions surrounding the central Ser/Thr phosphorylation site. However, as these peptides contain two phosphorylation sites, these strong signals may not reflect true selectivity. No strong positive or negative selections for phosphoamino acids (pThr or pTyr) were observed at any position.

**Figure 2.**
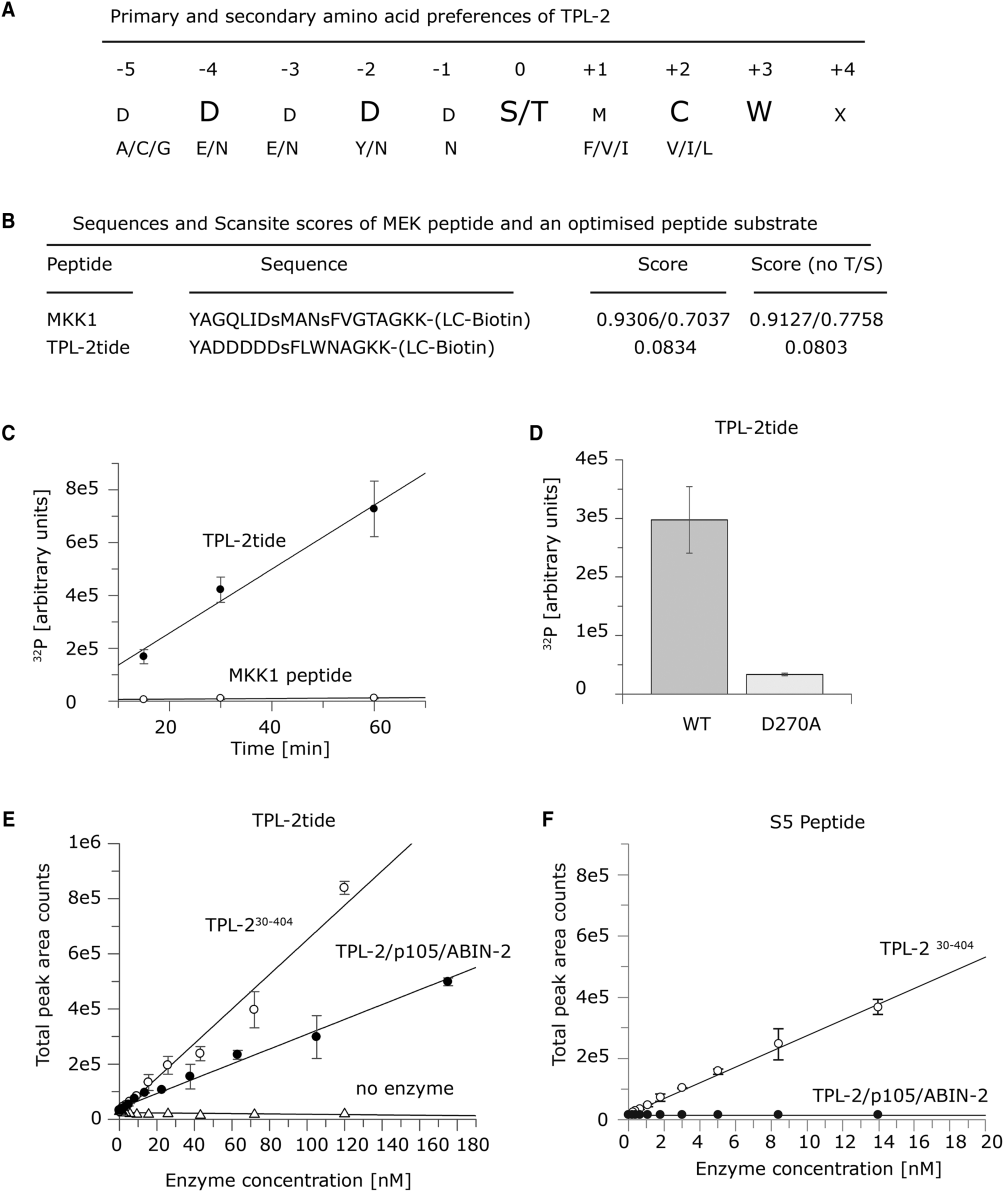
Testing an optimized peptide substrate for TPL-2/NF-κB1 p105/ABIN-2. (**A**) The primary and secondary amino acid preferences for phosphorylation by the recombinant TPL-2/NF-κB1 p105/ABIN-2 complex are shown. (**B**) The sequences and scansite scores for the MKK1 activation loop and optimized TPL-2tide peptide substrates. (**C**) Time-course experiment comparing TPL-2/NF-κB1 p105/ABIN-2 complex phosphorylation of MKK1 and TPL-2tide peptides (50 µM final concentration). Assays were performed with 30 nM the recombinant TPL-2/NF-κB1 p105/ABIN-2 complex in TPL-2 kinase buffer plus 1 mM ATP and 0.02 µCi/µL [γ-^32^P]ATP. Peptides were transferred onto streptavidin-coated membranes, which were then extensively washed. Incorporation of ^32^P into peptides was quantified by phosphorimaging. Linear regression was fitted with GraFit version 7.0.3. Values are means ± SD for three replicate reactions. (**D**) Kinase assays as in (**C**) comparing TPL-2tide peptide phosphorylation by wild-type (WT) or kinase-inactive (D270A) TPL-2/NF-κB1 p105/ABIN-2 complex (15 min). (**E** and **F**) TPL-2^30–404^ and TPL-2/NF-κB1 p105/ABIN-2 were titrated (1 : 1.66) in 384-well plates. A substrate solution containing ATP and TPL-2tide (**E**) or S5 peptide (**F**) was then added. The plates were analyzed using a Sciex API6500 (**E**) or API4000 (**F**) Triple Quad with RapidFire™ Technology. The graph shows total peak area counts for the MRM transition 953.6/904.3 Da (**E**; phosphorylated TPL-2tide peptide) and 612.9/120 Da (**F**; phosphorylated S5 peptide) for the indicated enzyme at increasing concentrations. Linear regression was fitted using GraFit software. In **E** and **F**, representative graphs from one experiment are shown, including standard deviation of triplicate assays.

The peptide library screen was repeated three times and selectivity values were calculated from spot intensities (Supplementary Table S1). Selectivity values quantitatively describe a kinase's preference for each amino acid at any one of nine positions surrounding the central phosphorylation site. These values can range from 0 (strongest negative selection) to 20 (strongest positive selection), where a value of 1.0 indicates no preference (i.e. neutral selectivity). Because of possible misinterpretation of preference for Ser/Thr, selectivity values were calculated to disregard positive selections of Ser or Thr and then combined into a PSSM. This analysis showed that TPL-2 exhibited comparatively weak positive selections, with Trp at P + 3 being the strongest preference with a selectivity value of 3.26. At other positions, TPL-2 generally tolerated several amino acids with similar physicochemical properties (Figure 2A). These data suggested that TPL-2 selects target residues to phosphorylate based on primary sequence properties, such as charge and hydrophobicity, rather than specific amino acid identity at positions relative to the phosphorylation site.

To test whether the peptide library screen could correctly predict phosphorylation efficiency of linear sequences by TPL-2, a synthetic peptide substrate was generated corresponding to an optimized sequence predicted by the TPL-2 consensus motif (Figure 2A). The sequence of this optimal peptide was modified, so that Phe and Leu residues replaced the reactive Met and Cys residues at P + 1 and P + 2, respectively. Because the selectivity values of these alternative amino acids were similar, these modifications were expected to have minimal effect on TPL-2 preference for this peptide. TPL-2 phosphorylation of optimized peptide substrate (termed TPL-2tide) was then compared with an MKK1 peptide control, using the recombinant purified TPL-2 complex (Figure 2B). TPL-2 complex phosphorylated TPL-2tide ∼60-fold more efficiently than MKK1 peptide at all time points (Figure 2C). TPL-2tide was minimally phosphorylated by kinase-inactive TPL-2[D270A] complex (Figure 2D), demonstrating that the measured kinase activity was indeed due to TPL-2. These results confirmed the TPL-2 specificity of the optimal phosphorylation site motif determined by peptide library screening.

### Development of a high-throughput kinase assay for the TPL-2 complex

Multiple screens by large pharmaceutical companies using the isolated TPL-2 kinase domain have identified small-molecule ATP-competitive inhibitors that specifically block TPL-2 signalling in intact cells, some of which also have activity in disease models in mice and/or rats [8,9]. However, none of these inhibitors have progressed to clinical trials, perhaps as a consequence of sub-optimal molecular properties or inadequate therapeutic index. Given the intensive resources previously dedicated to screening with the isolated TPL-2 kinase domain, a new screening approach, with the potential to expose more molecular interactions, may ultimately prove the best route to the development of anti-inflammatory drugs from this target.

Our earlier study has indicated that TPL-2 promotes the production of TNF in LPS-stimulated macrophages independently of ERK1/2 activation when still bound to NF-κB1 p105 and ABIN-2 [13]. The TPL-2/NF-κB1 p105/ABIN-2 complex is, therefore, believed to offer a more physiologically relevant target for the development of drugs to treat TNF-mediated inflammatory diseases. It is likely that the TPL-2 kinase domain adopts a different conformation when bound to NF-κB1 p105 and ABIN-2 than in isolation. In addition, the isolated TPL-2 kinase domain has a tendency to associate with heat shock proteins and is unstable, in contrast with the TPL-2/NF-κB1 p105/ABIN-2 complex [14,27]. Consequently, screens with the TPL-2 complex might identify different types of inhibitors with better properties for drug development than those identified with the isolated TPL-2 kinase domain. These could include non-ATP-competitive allosteric inhibitors, which might have better physicochemical properties. With this aim in mind, we developed a high-throughput assay to monitor phosphorylation of TPL-2tide by the TPL-2/NF-κB1 p105/ABIN-2 complex.

We used the RapidFire™ high-throughput mass spectrometry system (Agilent Technologies) to analyze TPL-2tide phosphorylation in a 384-well plate format. This label-free method directly measures phosphorylated product and is not commonly prone to interference seen in assays using fluorescent and absorbance readouts. MRM was used to simultaneously detect unphosphorylated and phosphorylated peptide. Owing to relatively low substrate conversion, peak area counts for the phosphorylated peptide were used to measure TPL-2 activity rather than percentage of substrate conversion from unphosphorylated to phosphorylated peptide. After 30 min incubation of reaction mixes, TPL-2tide phosphorylation increased with increasing complexed TPL-2 enzyme concentration. The isolated TPL-2 kinase domain, TPL-2^30–404^ [9], was also able to phosphorylate TPL-2tide, with slightly higher efficiency than complexed TPL-2. A large signal-to-noise ratio was achieved with both complexed TPL-2 and TPL-2^30–404^ with enzyme concentrations at 80 nM, which was used for subsequent inhibitor assays (Figure 2E). A *Z*-factor of 0.6–0.7 indicated that this assay was robust and suitable for high-throughput screening.

Comparative analysis of synthetic MEK-1 and phospho-MEK1 peptides demonstrated that the RapidFire™ assay was capable of detecting MEK1 peptide phosphorylation (data not shown). However, we were unable to detect phosphorylation of an MEK1 activation loop peptide with this assay, using either complexed TPL-2 or TPL-2^30–404^. Using the TPL-2 PSSM, Scansite 2.0 determined whether MKK1 peptide exhibited similarity to the optimal TPL-2 complex consensus motif. Phosphorylation sites in the activation loop of MKK1/2 generated only scored just below 1.00 (0.78 and 0.91), with a score of 1.0 indicating no selection. This suggests that TPL-2 does not select MEK1 as a substrate based on its linear amino acid sequence. Rather, other mechanisms such as protein–protein interaction are likely involved. Consistent with this hypothesis, TPL-2 binds to MEK1 protein and this interaction is blocked by p105, which inhibits TPL-2 MEK1 kinase activity [10].

S5 (GAFRSAIRRLAARRR), an optimized peptide substrate for the isolated TPL-2 kinase domain identified by IMAP peptide library screen, was not phosphorylated by complexed TPL-2 (Figure 2F). In contrast, TPL-2^30–404^ phosphorylated S5 peptide efficiently, as expected. These data support the hypothesis that complexed TPL-2 does not have the same substrate specificity as the isolated TPL-2 kinase domain. The isoelectric point of the S5 peptide is 12.54, compared with 3.74 for TPL-2tide, suggesting that peptide substrate selection by the isolated TPL-2 kinase domain and TPL-2 complex is dependent on the charge of the amino acids surrounding the phosphorylated serine.

### Differential sensitivity of complexed TPL-2 and TPL-2 kinase domain to TPL-2 inhibitors

**Figure 3.**
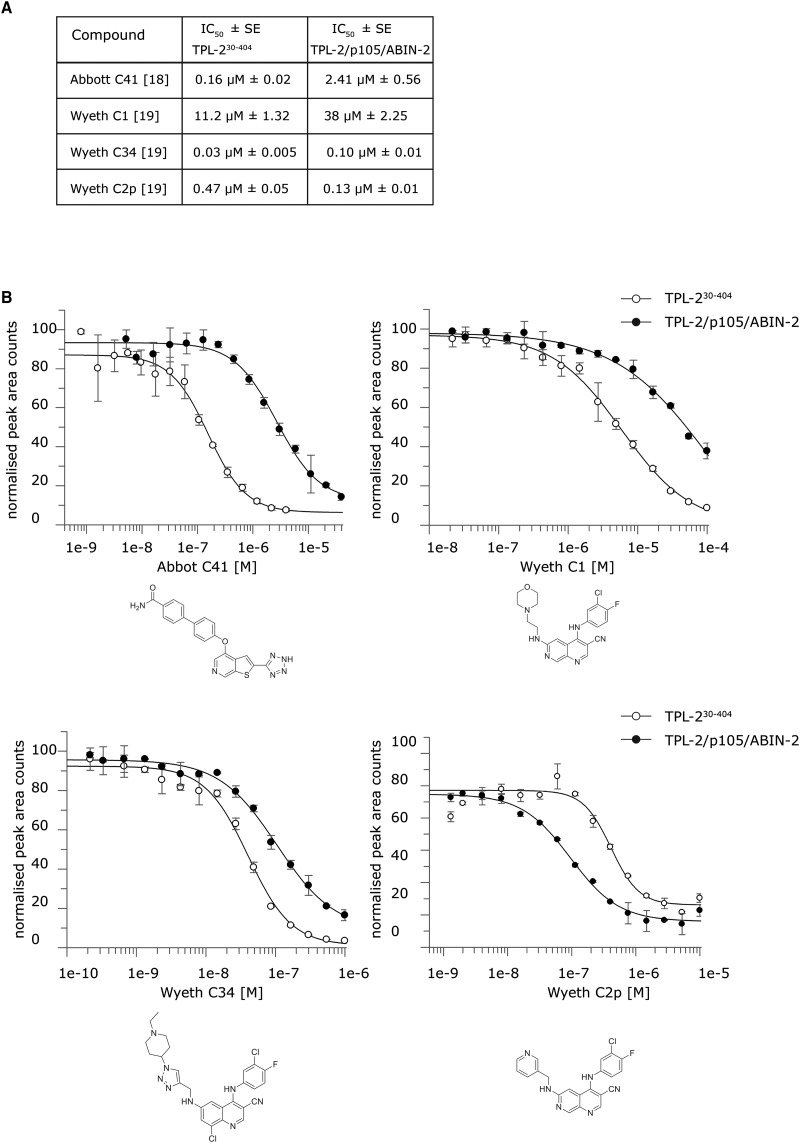
High-throughput Rapid Fire™ mass spectrometry assay of TPL-2/NF-κB1 p105/ABIN-2 complex kinase activity. (**A**) The efficacy of known TPL-2 inhibitors was tested for TPL-2^30–404^ and the TPL-2 complex. 5 μL of the enzyme solutions were added to each well (two replicates for each using final concentrations of 80 nM TPL2 30–404 and 80 nM TPL-2/NF-κB1 p105/ABIN-2). The compounds were then dispensed using a HP digital dispenser. The enzyme/compound mixture was incubated for 10 min before the addition of 5 µL of substrate buffer containing final concentrations of 100 µM TPL-2tide and 400 µM ATP to initiate the reaction. The reactions were incubated for 30 min quenched with 50 µL of 1% formic acid. The plates were analyzed using a Sciex API6500 Triple Quad with RapidFire™ Technology. The data was analyzed using product counts for the MRM transition 953.6/904.3 Da (phosphorylated peptide) using a four-parameter logistical equation fitted using GraFit software to determine IC_50_ values. For each compound, multiple replicates were run for each experiment and the experiment was repeated four to five times. The IC_50_ values shown are generated from all datasets analyzed and include standard errors (SE). (**B**) Representative graphs of one experiment for each compound are shown, including standard deviation from replicates.

Wyeth (Pfizer), Abbott and Novartis have each run large inhibitor screening programmes targeting the kinase domain of TPL-2 [8,9,17,28]. This has resulted in many ATP-competitive small-molecule inhibitors, which block TPL-2 catalytic activity *in vitro* and in cells. We next tested the sensitivity of the TPL-2 complex to a small panel of these inhibitors in comparison with TPL-2^30–404^ kinase domain by measuring TPL-2tide phosphorylation over a range of compound concentrations (Figure 3A,B).

Complexed TPL-2 was substantially less sensitive to inhibition by Wyeth C1 [18,20] and Abbott C41 [19]. Wyeth C34 [21] also inhibited TPL-2[30–404] slightly more efficiently than complexed TPL-2. In contrast, Wyeth C2p [18,20] more potently inhibited complexed TPL-2 than TPL-2^30–404^. The significant differences in sensitivities of complexed TPL-2 and TPL-2^30–404^ to ATP-competitive inhibitors may indicate that the ATP-binding pocket of TPL-2 associated with NF-κB1 p105 and ABIN-2 adopts a different conformation to that in TPL-2^30–404^. Consistent with this, the TPL-2 kinase domain forms interactions both with NF-κB1 p105 and its own C-terminus [10,29], which is lacking in TPL-2^30–397^ and TPL-2^30–404^ used in earlier inhibitor screens.

TPL-2 kinase is a promising drug target for the treatment of inflammatory diseases due to its critical role in regulating the production of TNF and its requirement for the development of inflammation in multiple mouse disease models [5]. Earlier screens for TPL-2 inhibitors have used the isolated TPL-2 kinase domain (TPL-2^30–397^ and TPL-2^30–404^) purified from baculovirus-infected insect cells and MKK1 peptide or protein substrates [8,9]. Notably, this approach has failed to generate any inhibitors that have progressed to clinical trials. The present study suggests that screening with TPL-2/NF-κB1 p105/ABIN-2, using the TPL-2tide substrate, has the potential to identify novel ATP-competitive TPL-2 inhibitor series that preferentially inhibit the kinase domain of complexed TPL-2 in comparison with the isolated TPL-2 kinase domain. In addition, it is possible that screening with the TPL-2 complex will identify allosteric inhibitors (that block TPL-2 signalling independently of binding to the TPL-2 ATP-binding site) that have increased specificity compared with ATP-competitive inhibitors. Furthermore, novel TPL-2 inhibitors identified in screens with the TPL-2/NF-κB1 p105/ABIN-2 complex may have improved *in vivo* potency that allows their development through to clinical trial as anti-inflammatory drugs.

## Abbreviations

DM: decyl β-d-maltopyranoside
ERK1/2: extracellular signal-regulated kinase 1/2
ESI: electrospray ionization
HA: haemagglutinin
IKK: IκB kinase
LPS: lipopolysaccharide
MAPK: mitogen activated protein kinase
MKK1: mitogen activated protein kinase kinase 1
MRM: multiple reaction monitoring
PSSMs: position-specific scoring matrices
TCEP: tris(2-carboxyethyl)phosphine
TLRs: Toll-like receptors
TNF: tumour necrosis factor
TPL-2: tumour progression locus 2
