# Supplementary material for: Assaying kinase activity of the TPL-2/NF-κB1 p105/ABIN-2 complex using an optimal peptide substrate
Source: Biochem J. 2018 Jan 11;475(1):329–40. doi: 10.1042/BCJ20170579 (PMC5763956; doi:10.1042/BCJ20170579)
Supplement: Supplementary Table S1 [file BCJ-475-329-s2.pdf]

Supplementary Table 1. TPL-2 position-specific scoring matrices generated by peptide library

Full motif

	**P**	**G**	**A**	**C**	**S**	**T**	**V**	**I**	**L**	**M**	**F**	**Y**	**W**	**H**	**K**	**R**	**Q**	**N**	**D**	**E**
**-5**	1.12	1.16	1.43	1.24	1.35	0.77	0.74	0.43	0.56	0.66	0.81	0.87	1.23	1.03	0.63	0.76	1.01	1.25	1.59	1.34
**-4**	1.04	1.31	1.29	0.87	1.43	0.86	0.47	0.48	0.56	0.76	0.54	0.9	1.08	1.18	0.51	0.45	1.08	1.46	2.15	1.58
**-3**	1.06	1.02	0.89	0.44	1.55	1	0.67	0.53	0.66	0.9	0.48	1.28	1.5	0.82	0.44	0.39	1.11	1.47	1.97	1.84
**-2**	0.57	0.85	0.82	0.48	1.04	0.93	0.51	0.66	1.21	0.98	1.16	1.67	1.35	0.78	0.31	0.24	0.9	1.45	2.71	1.36
**-1**	1.05	0.54	0.75	0.26	1.28	0.94	0.71	0.7	1.23	1.22	0.78	1.29	1.09	1.17	0.93	0.65	1.29	1.55	1.58	0.99
**0**																				
**+1**	0.23	0.6	0.64	0.58	1.78	1.34	1.49	1.67	1.12	1.78	1.5	1.39	1.1	0.8	0.38	0.31	1.14	1.03	0.56	0.56
**+2**	0.72	1.11	0.93	1.97	1.14	0.85	1.14	1.25	1.41	1.05	0.95	1.14	1.21	1.11	0.5	0.48	0.99	1.17	0.47	0.41
**+3**	0.9	1.11	0.9	0.64	1.57	1.07	0.86	0.79	1.05	1.14	0.96	1.4	3.14	1.15	0.41	0.42	0.88	0.97	0.4	0.24
**+4**	1.02	1.35	0.97	1.14	1.1	1.22	1.03	0.72	0.86	1.09	0.92	0.87	1.06	1.2	0.62	0.38	1.17	1.35	1.08	0.87

No Ser/Thr

	**P**	**G**	**A**	**C**	**S**	**T**	**V**	**I**	**L**	**M**	**F**	**Y**	**W**	**H**	**K**	**R**	**Q**	**N**	**D**	**E**
**-5**	1.15	1.19	1.46	1.26	1	0.79	0.75	0.44	0.57	0.67	0.83	0.89	1.25	1.05	0.65	0.78	1.03	1.28	1.61	1.37
**-4**	1.07	1.34	1.32	0.89	1	0.88	0.48	0.49	0.58	0.77	0.55	0.92	1.11	1.21	0.52	0.46	1.11	1.49	2.2	1.61
**-3**	1.09	1.05	0.92	0.46	1	1	0.69	0.55	0.68	0.92	0.49	1.32	1.54	0.84	0.46	0.4	1.14	1.51	2.04	1.9
**-2**	0.57	0.85	0.83	0.48	1	0.94	0.51	0.67	1.21	0.99	1.16	1.68	1.35	0.78	0.31	0.24	0.9	1.45	2.72	1.37
**-1**	1.07	0.55	0.76	0.27	1	0.95	0.72	0.72	1.25	1.24	0.79	1.31	1.1	1.19	0.94	0.66	1.31	1.57	1.6	1.01
**0**																				
**+1**	0.25	0.64	0.68	0.62	1	1	1.59	1.79	1.19	1.9	1.6	1.48	1.18	0.86	0.4	0.33	1.21	1.1	0.6	0.59
**+2**	0.72	1.12	0.93	1.98	1	0.85	1.15	1.26	1.42	1.06	0.96	1.15	1.22	1.12	0.5	0.48	1	1.18	0.48	0.42
**+3**	0.93	1.15	0.93	0.66	1	1	0.89	0.82	1.09	1.18	0.99	1.45	3.26	1.19	0.43	0.43	0.91	1.01	0.42	0.25
**+4**	1.04	1.38	0.98	1.16	1	1	1.05	0.74	0.88	1.11	0.94	0.88	1.07	1.22	0.63	0.38	1.19	1.38	1.09	0.89
